# Factors in an Auto-Brewery Syndrome group compared to an American Gut Project group: a case-control study

**DOI:** 10.12688/f1000research.52743.1

**Published:** 2021-06-08

**Authors:** Barbara Cordell, Anup Kanodia, Gregory K. Miller

**Affiliations:** 1 Department of Nursing, Panola College, Carthage, TX, 75633, USA; 2 Ohio State University Medical Center, Columbus, OH, 43210, USA; 3 Department of Statistics, Stephen F. Austin State University, Nacogdoches, TX, 75962, USA

**Keywords:** Auto-Brewery Syndrome, Gut Fermentation, Yeast Overgrowth, American Gut Project, Microsetta, Endogenous Alcohol

## Abstract

**Background: **Auto-Brewery Syndrome (ABS), also known as Gut Fermentation Syndrome, is a rare but underdiagnosed condition. While scores of case studies of ABS are published, only one previous study examined ABS patients’ demographics, health history, lifestyle factors, and diet compared to a control group of household members.

**Methods:** We designed a case-control study to identify factors that individuals with a diagnosis of ABS and those who live with them might have that differ from a larger general group. We administered a survey to 46 patients known to have a diagnosis of ABS and their household members. Here, we compare our group of survey takers to a cohort of the American Gut Project (AGP) participants (N=11,297) for the 30 questions that were identical.

**Results:** With a response rate of 88% and using Rank Sum Tests, the data demonstrate that patients with ABS and their household members are more likely than participants of the AGP to own a pet (p=.03 for cat; p=.0001 for dog), get less sleep (p=.0001), and have lesser quality of bowel movements (p=.03). In addition, the ABS group consumes more water (p=.02) and less alcohol (p=.0004), eats at home more often (p=.0056), and reports more aversion to sweets (p=.01). The most striking difference is a higher presence of non-food allergies in all five subcategories of the survey in the ABS group compared to the AGP group.

**Conclusion:** Patients with ABS and their household members show several significant differences in their lifestyle and health, diet, and medical history compared to a large group of AGP participants. These differences lead to several hypotheses about co-morbidities that warrant further research.

## Introduction

Auto-Brewery Syndrome (ABS), also known as Gut Fermentation Syndrome and ‘endogenous ethanol fermentation’, is a rare but underdiagnosed syndrome ^1^. An overgrowth of fermenting yeast or bacteria in the intestines causes patients to ferment carbohydrates and sugars into ethanol. Patients test positive for blood alcohol, often at very high levels, without ingesting alcohol but may or may not exhibit signs and symptoms of intoxication.

The earliest cases were reported in France in 1894 and translated from French into English in 1906 in the book *Auto-Intoxication in Disease* ^2^. Since then, at least 68 case studies in Japan, Canada, the United States, Italy, and other countries were published in the post-1950s academic literature ^3^. Of those, 14 individual cases have been published in the last decade and the disease is finally starting to be recognized as a rare but treatable syndrome ^4^.

We observe that ABS is often reported in people with co-morbidities, such as gastroparesis, Crohn’s disease, short bowel syndrome, and obesity-related liver disease ^5, 6^ but otherwise-healthy people are being diagnosed as well ^7, 8^. While numerous case studies describe the diagnosis and treatment, little is known about the factors that cause or even contribute to ABS.

The scores of published cases of ABS, with two exceptions, all point to an over-colonization of fermenting yeast in the gastrointestinal tract. The first anomaly is the case of a man in China with severe non-alcoholic steatohepatitis and bacterial ABS ^9^. Strains of a high alcohol-producing *Klebsiella pneumoniae (HiAlc KPN)* were strongly associated with his endogenous production of alcohol. With further research, the *HiAlc KPN* was associated with 60% of people with non-alcoholic fatty liver disease (NAFLD) in a cohort of Chinese people. Furthermore, isolates of the microbes transferred from the subjects to healthy mice induced NAFLD. The second deviation from the norm is a case study published with the location of the fermenting yeast, *Candida glabrata*, in the patient’s urinary system ^10^. The authors demonstrated high levels of ethanol production in the patient’s urine that was confirmed with an in vitro experiment.

Of the thousands of different species of microbes in an individual’s gut, an estimate of less than 0.1% are considered rare, and fungi belong to the rare biosphere known as the mycobiome. Most of the reports of fungal infections focus on yeast and fungal overgrowth in immunocompromised patients but the incidence of fungemia is increasing. Symptoms of yeast overgrowth include fatigue, headaches, poor concentration, gastrointestinal distress, muscle pain, joint pain, and urogenital symptoms ^11^.

We now know that underlying genetic susceptibility to *Candida* plays an important role in infections. Normally, natural defense mechanisms prevent the commensal *Candida* and other yeasts from becoming pathogenic. But loss of barrier function, imbalances in the gut microbiome, or inadequate immunity may lead to increased susceptibility to invasive candidiasis ^12^.

In addition to the universally demonstrated overgrowth of fermenting yeast or bacteria, patients with ABS often have signs of yeast infections in their integumentary, genitourinary, or oral systems. Some patients with ABS respond to dietary changes alone, while others require anti-fungal medication, dietary changes, supplements, probiotics, and lifestyle modifications such as adequate sleep, stress-reduction, and moderate exercise. A small number of patients do not seem to respond to any of the mentioned treatments or will persistently relapse. For these chronic patients, a Fecal Microbiota Transplant (FMT) appears to be a promising treatment. A patient in Belgium received what we believe is the first published case of a successful FMT for ABS ^13^.

Of four previous studies on groups of fermenting patients, only one looked at ABS patients’ demographics, health history, lifestyle factors, and diet to determine factors that might contribute to ABS ^14^. The study demonstrates that patients with ABS or symptoms have significant differences in their lifestyle and health, diet, and past medical history compared to household participants who live with someone with ABS but do not have clinical ABS.

The purpose of the current research project is to identify the health history and lifestyle factors of subjects diagnosed with ABS and their household members and compare those factors in subjects of a large control group from the database of the American Gut Project (AGP). The AGP is a crowd-funded science project to analyze the gut microbiomes of a large cross section of American citizens and determine what health and lifestyle factors correlate to different microbiomes ^15^. The null hypothesis is that there will be no differences between the ABS and household members compared to the AGP group on thirty questions related to health and lifestyle.

## Methods

### Study design

We designed a case-control study in 2016 to identify factors that individuals with a diagnosis of gut ABS and those who live with them might have that differ from a more general group in the AGP. We developed a survey that included 30 questions identical to those used in the AGP group and could be easily administered online. The 30 identical questions were used for analysis in this study. Our study design was submitted to and approved by the Institutional Review Board at Panola College in Carthage, TX, USA with approval number PC-IRB-08032016.

### Survey

A 78-item survey titled, *Gut Fermentation Survey*, was developed using questions from the AGP as well as questions from other well-known survey sources detailed below. In addition to questions for demographics and diagnosis, we used 30 of the AGP questions and these comprise the questions for the current statistical analysis between the two groups. The AGP supplied us with their questionnaire and permission to use any of their questions. In addition, they supplied data on 11,297 respondents who also answered the 30 questions as they participated in a volunteer study that used a longer questionnaire and collected stool samples ^16^. Bias is minimized in the questionnaire by using questions from well-known questionnaire sources that have validity and reliability.

Questions 1–8 are demographic questions patterned after the Integrated Postsecondary Education Data System ^17^ from the National Center for Education Statistics (NCES) of the Department of Education. Questions 9–11 ask about diagnosis and symptoms of ABS and serve to divide the group of respondents into the ABS group and those who live with someone with ABS. Questions 12–37 query Lifestyle and Health factors such as living arrangements, dental care, tobacco use, bowel habits, sleep habits, and pet ownership. The tobacco questions come from the World Health Organization ^18^. The bowel habit questions are from the AGP, and the sleep questions are from the Mini-Sleep Questionnaire ^19^.

Questions 38–55 ask about General Diet Information such as type of diet followed, amount and type of liquids consumed, alcohol consumption, and food allergies. The dietary questions are from the AGP except for the questions on alcohol use that came from the National Institute on Alcohol Abuse and Alcoholism ^20^. Questions 56–72 concern the individual’s Health History such as the method of birth, whether the subject was breast-fed or given formula and how long, past surgeries, medical conditions past and present, as well as non-food allergies. The questions on breastfeeding came from the survey methods of breastfeeding from the Centers for Disease Control and Prevention ^21^ and the other questions are from the AGP. Questions 73–77 inquire about medications and supplements and question 78 asks if the respondent would be willing to be contacted concerning future research opportunities. Information on access to the questionnaire is provided at the end of the article.

### Participants

Between September 2016 and January 2017, we emailed requests to participate in the study to people known to the authors to either have a diagnosis of ABS or share a household with a person diagnosed with ABS. A hyperlink to the survey was included in the email request. Because ABS is very rare, we invited everyone we knew from various medical practices treating ABS, from a Facebook support group for ABS, as well as from emails sent to the author from her website on ABS. Diagnosis was confirmed by the diagnosing provider or a medical record. A $25 Amazon gift card was offered as an incentive to those who completed the survey. Bias was minimized by using a standard email message, one person to answer questions, and a uniform informed consent.

In total, all 52 people known to the authors to have ABS or to live with someone with ABS were invited to participate in the study. People with ABS were included if they were at least 18 years of age and had been formally diagnosed by a professional licensed to diagnose in their state or country. Household members were included if they lived in the same house as a person diagnosed with ABS and were at least 18 years of age. Potential participants were excluded if they were under 18 or unable to complete a survey, even with assistance. Of those invited, 46 agreed to participate, received the informed consent document, and completed the electronic *Gut Fermentation Survey* for an 88% response rate. The informed consent was included as the page prior to the survey and subjects were told that proceeding with the survey served as their consent. Of the 46 respondents, 28 had a history of ABS and the other 18 respondents lived in the same dwelling as one of the people with ABS. Hereinafter, the ABS and household groups are combined and labeled as the ABS group that comprised the group we compared to the data from the AGP.

### Statistical analysis

The statistical analysis compared the answers for the 30 questions from the ABS and household respondents (N=46) to the answers on identical questions administered by the AGP survey (N=11,297). For those 30 questions on the two surveys, 2 by *c* contingency tables were created to tabulate question responses and test for row homogeneity (ABS vs. AGP group) using a permutation chi-square test.

All such permutation chi-square tests were performed using *StatXact* Version 4.0.1. Additional permutation *Kruskal-Wallis* tests reduced to Rank Sum Tests (also known as *Mann-Whitney Wilcoxon Rank Sum Tests*) were used to analyze row homogeneity between the ABS and AGP groups in the cases where survey questions generate ordinal responses.

We note that with such a large sample size in the control group from the AGP, our focus is less on p-values and more on the actual differences (observed effect sizes) seen in the percentages. These are provided in subsequent sections.

## Results

### Demographics

The demographics of the 46 ABS participants are detailed in Table 1. All participants were over the age of 18 as verified in the informed consent. The majority of participants were White, not Hispanic or Latino, and were born and lived most of their lives in the United States.

**Table 1.** Demographics of respondents.

Group	ABS and household members (N = 46)
Sex	Female = 26; Male = 20
Continent where subject was born	1 = Africa
	2 = Europe
	1 = South America
	42 = United States
Continent where subject lived most of life	1 = Europe
	1 = Africa
	44 = United States
Race	2 = American Indian or Alaskan Native
	1 = Black or African American
	2 = Asian
	41 = White
Ethnicity	3 = Hispanic or Latino
	2 = Did not answer
	41 = Not Hispanic or Latino

### Data analysis of 30 questions

*Lifestyle and Health:* In the *Lifestyle and Health* section of the *Gut Fermentation Survey*, there are three statistically significant differences between the responses from the ABS group and those in the AGP group. Data show that those in the ABS group are: (1) more likely to own a traditional pet (41% vs. 27%, *p =* .03 for cat, 57% vs. 29%, *p = .*0001 for dog); (2) less likely to get 7 hours or more of sleep per night on average (31% vs. 59%, *p* = .0001); and (3) less likely to report typically having normal bowel movements (55% vs. 72%, *p* = .03) than those in the AGP group.

*General Diet Information:* Regarding dietary intake, those in the ABS group tend to report a higher tendency to typically consume at least one liter of water per day (62% vs. 50%, p = .02). The ABS group tends to consume less alcohol than the AGP group with a higher percentage reporting complete abstinence (47% vs. 24%, p = .0004). We see a difference in the distribution of frequency of eating out between the two groups (p = .0056) with 16% of ABS respondents stating they never eat out compared to 8% for the AGP participants. The ABS group also stated an aversion to sweets with 36% stating they refrain compared to 13% in AGP group (p = .01).

*Health History:* A sweeping difference between the ABS and AGP group is seen concerning the presence of allergies. The ABS group is far more of an allergic group than the AGP respondents. We see a theme where for each subcategory of non-food allergy, the ABS group shows a higher presence of an allergic reaction. Table 2 details the five subcategories of allergies with the comparison of the ABS group percentages to those of the AGP group. P-values for each comparison resulting from the associated 2 by 2 contingency table are provided. The ABS group was more likely to have seasonal allergies, drug allergies, and pet dander allergies. They were also more likely to have an allergy to bee or wasp stings and poison ivy or poison oak.

**Table 2.** Allergies in the Auto-Brewery Syndrome group vs. American Gut Project group.

Type of allergy	Comparison	P-value
Seasonal Allergies	More likely for an ABS respondent to have seasonal allergies (60%) compared to AGP respondents (40%)	0.0062
Drug Allergies	More likely for an ABS respondent to have drug allergies (25%) compared to AGP respondents (9%)	0.0025
Pet Dander	More likely for an ABS respondent to have allergy to pet dander (17%) compared to AGP respondents (6%)	0.0112
Bee Stings	More likely for an ABS respondent to have allergy to bee/wasp stings (9%) compared to AGP respondents (2%)	0.0076
Poison Ivy/Oak	More likely for an ABS respondent to have allergy to poison ivy/oak (24%) compared to AGP respondents (7%)	0.0002

As telling as the significant results are, the items without statistical significance are still important to consider. Much research about how the microbiome develops and differs between cultures has to do with factors such as the method of birth (vaginal vs. cesarean), infant feeding (breast vs. formula), and childhood illnesses such as chickenpox. Even though the data show no significance on these three items between the ABS group and the AGP group, these factors should not be dismissed but be examined further. Perhaps a larger group of people with ABS would reveal different results.

Issues of immunity such as presence or absence of tonsils and appendix, and history of flu vaccine were also assessed, and the lack of significant differences does not mean those factors do not influence ABS

## Discussion

Using the 30 identical questions from the *Gut Fermentation Survey* and AGP survey, we found 12 statistically significant differences between people with ABS or living with someone with ABS and the more general group of persons participating in the AGP.

In the *Lifestyle and Health* section, the fact that people in the ABS group are more likely to have a lesser quality of bowel movements than the large data set (N=11,297) from the AGP, supports the idea that gut dysbiosis is the underlying cause of ABS. This a similar finding to previous research we conducted and strengthens the finding ^14^. People with perturbations in the gut microbiota would be expected to have changes in bowel habits and conversely changes in bowel habits reflect gut dysbiosis. There is evidence that changing the gut microbiota through fecal transplant, often relieves symptoms such as diarrhea, indigestion, and abdominal pain ^22^.

The strongly significant difference that subjects in the ABS group get less sleep than the control group may indicate there are factors of ABS such as intoxication, that cause patients to lose sleep. A study of 393 participants who visited a hospital demonstrated a high correlation in men between alcohol use and sleep disturbances as well as the perception of poor quality of sleep ^23^. People who generate their own alcohol would likely suffer the same ill effects.

The *General Diet Information* section provides data similar to previous research in that people with ABS consume more water and less alcohol. In addition, they are less likely to eat meals away from home and more likely to avoid sweets. *Candida* overgrowth in the gut is associated with inflammation and other gastrointestinal diseases such as Crohn’s disease, ulcerative colitis, and gastric ulcers, which can lead to painful symptoms and loss of appetite ^24^.

These findings could also be due to the dietary changes made by patients who have become aware of dietary triggers and are making informed decisions to change their eating habits. Additional information should be collected on these and other patients to determine what dietary habits they had historically, versus the dietary patterns adopted once they became aware of ABS symptoms, triggers, and treatments.

The most interesting segment of the survey comparison is the *Health History* section. The participants in the ABS group are much more allergic overall than the 11,297 respondents of the AGP. The ABS group is more likely to have seasonal allergies, have drug allergies, be allergic to pet dander, be allergic to bee stings, and be allergic to poison ivy or poison oak. Overall, people who suffer from ABS and those who live with them are a much more atopic group than those in the AGP. These data suggest a possible link to immune systems that may be interacting with gut dysbiosis in the expression of symptoms.

Food sensitivities are closely related to other types of allergic reactions. Fermentable carbohydrates termed ‘FODMAPs’ (fermentable oligo- di- mono-saccharides and polyols) occur in many foods and are associated with many gastrointestinal symptoms such as IBS. Such sensitivities and even intolerances have been linked to eczema, asthma, food allergies, and allergic rhinitis ^25^.

People with ABS have gut dysbiosis by the very presence of an over-colonized fermenting yeast or bacteria and by the production of endogenous alcohol. Symptoms of gut fermentation may mimic food sensitivities and it would make sense that fermentable carbohydrates would create additional gastrointestinal symptoms. The fact that the ABS group is more allergic follows the theory of ‘allergic march’ where there are patterns of co-morbidities of allergic disease and even progression rather than a single disease ^26^. This hypothesis should be explored further.

The significant difference that people in the ABS group are more likely to own a pet, indicates animals could be a possible contributing factor. Even though people with ABS are more likely to be allergic to pet dander, they are also more likely to own a pet. This raises the question of whether owning a pet further exacerbates fermentation and symptoms. And it also raises the question whether an allergy to pet dander contributes somehow to gut fermentation.

One limitation to the study is the online nature of the survey. Not all potential participants have access to a computer or are willing to take an online survey. These people may differ from those who do have a computer and are willing to take an online survey. Another limitation to the study is the small sample size. Significance from other questions may not have been detected with such a small ABS sample. The lack of significant results in the factors mentioned above leads us to believe these factors are not related to the development of ABS but they might still be important in future studies. While we certainly need to focus in on the significant differences, we should dig deeper into other factors to see how ABS develops. An important study would be to look at these and other factors in patients when they are first diagnosed prior to treatment.

Our ABS sample size is seemingly small but considering the rarity of ABS, we felt fortunate to have as many participants as we did. Because our analysis contained patients from four continents and our response rate was 88%, we are confident in generalizing our results to the growing field of ABS.

## Conclusion

Auto-Brewery Syndrome is a rarely diagnosed syndrome affecting people worldwide. While many case studies have been published there is scant research on ABS. Very little is known about lifestyle factors such as health, diet, and past medical history of ABS patients that may contribute to symptoms. Using questions from the nationwide AGP survey, our research showed 12 statistically significant differences between the group of ABS patients and housemates, and the more general group from the AGP.

The findings in this case control study give insights into common factors experienced by people with ABS and their household members. We believe our findings raise interesting hypotheses about co-morbidities. These hypotheses warrant additional research into influences that may impact the microbiome and lead to ABS. We recommend that providers in emergency departments and gastroenterology practices retain an open mind when they meet intoxicated patients who insist they have not been drinking. The more we learn about ABS the more we can respond with compassion and science-based information.

## Data availability

### Underlying data

Dryad: ABS Questionnaire 2016 and Questionnaire results 2018, https://doi.org/10.5061/dryad.vq83bk3sg ^27^.

- This project contains the survey responses from the 46 participants for the 78 questions of the *Gut Fermentation Survey.*
- Data are available under the terms of the Creative Commons Zero "No rights reserved" data waiver (CC0 1.0 Public domain dedication).

Qiita: American Gut Project – ID 10317, Accession number 10317: https://qiita.ucsd.edu/study/description/10317#

- This project contains the survey responses from the 11,297 American Gut Project (AGP) participants prior to 2016.
- Qiita is free to use but requires the registration before access. Users can register for free here.

European Nucleotide Archive: American Gut Project. Accession number ERP012803, https://www.ebi.ac.uk/ena/browser/view/PRJEB11419

### Extended data

Dryad: datadryad.org, ABS Questionnaire 2016 and Questionnaire results 2018, https://doi.org/10.5061/dryad.vq83bk3sg ^27^.

- This project contains the survey sent to participants
- Data are available under the terms of the Creative Commons Zero "No rights reserved" data waiver (CC0 1.0 Public domain dedication).
