# Supplementary material for: Factors in an Auto-Brewery Syndrome group compared to an American Gut Project group: a case-control study
Source: F1000Res. 2021 Jun 8;10:457. [Version 1] doi: 10.12688/f1000research.52743.1 (PMC8422346; doi:10.12688/f1000research.52743.1)
Supplement: Peer review history

# Peer review: Reviewer response for version 1

**DOI:** 10.5256/f1000research.56059.r89069

**Published:** 2021-09-16

**Authors:** Gaku Takahashi

**Affiliations:** 1 Iwate Medical University, Marioka, Japan

I had a very interesting look at the relationship between homebrewing syndrome and allergic constitution. I have read your treatise and have some questions. A recent treatise suggested a link between homebrewing syndrome and alcohol-degrading enzymes. Therefore, in this study as well, I think it is necessary to further investigate the strength of individual reactions to alcohol. It may not be possible to conduct a survey at the genetic level, but I think an additional questionnaire survey is necessary. In addition, I think it is necessary to list all the items and results of the questionnaire survey in the table. Please consider these points.

 I have some further questions, so please consider and improve. From the results of the questionnaire, it was said that there is a relationship between autobrewing syndrome and allergies. I think it is necessary to consider the reason for this. I also speculate that autobrewing syndrome is associated with alcohol-degrading enzyme function, periodontal disease, and gut microbiota. If possible, please consider the constitution of each case with alcohol and the presence or absence of periodontal disease. Please consider referring the following paper of mine. Gaku T, *et al*. Auto-brewery syndrome caused by oral fungi and periodontal disease bacteria. Acute med Surg.

Is the work clearly and accurately presented and does it cite the current literature?

Yes

If applicable, is the statistical analysis and its interpretation appropriate?

Yes

Are all the source data underlying the results available to ensure full reproducibility?

Yes

Is the study design appropriate and is the work technically sound?

Yes

Are the conclusions drawn adequately supported by the results?

Yes

Are sufficient details of methods and analysis provided to allow replication by others?

Partly

Reviewer Expertise:

Infection

I confirm that I have read this submission and believe that I have an appropriate level of expertise to confirm that it is of an acceptable scientific standard, however I have significant reservations, as outlined above.

# Peer review: Reviewer response for version 1

**DOI:** 10.5256/f1000research.56059.r87176

**Published:** 2021-09-06

**Authors:** Jing Yuan

**Affiliations:** 1 Department of Bacteriology, Capital Institute of Pediatrics, Beijing, China

Auto-brewery syndrome (ABS), also named gut fermentation syndrome, drunkenness disease or endogenous ethanol fermentation, is a syndrome in which patients become intoxicated without ingesting alcohol. As a rare case, the pathogenesis and inducement of ABS are not fully understood. In this study, the author investigated the health history and lifestyle factors of subjects between ABS and control group (AGP). As most of the ABS cases could be control through dietary control and lifestyle changes, the design of the subject can help us to understand the disease induce factors of the ABS group, so as to prevent the development. They found that ABS group are more likely than to own a pet, get less sleep, and have lesser quality of bowel movements. In addition, the ABS group consumes more water and less alcohol, eats at home more often, and reports more aversion to sweets.

 This study was well designed and the result has certain reference significance to the following research for ABS. The manuscript is well written and attractive, but I have some minor concerns on diet Information. They showed that ABS group more likely to avoid sweet. But the onset of most ABS cases was reported to consuming more carbohydrate, especially sugar. The author explained that this may due to the dietary changes made by patients who have become aware of dietary triggers and are making informed decisions to change their eating habits. I recommended to add more detail questions in this part, like the diet before and after ABS diagnosis. It will have a good help for clinician and epidemiologist to understand the dietary triggers for ABS.

Is the work clearly and accurately presented and does it cite the current literature?

Yes

If applicable, is the statistical analysis and its interpretation appropriate?

Yes

Are all the source data underlying the results available to ensure full reproducibility?

Yes

Is the study design appropriate and is the work technically sound?

Yes

Are the conclusions drawn adequately supported by the results?

Yes

Are sufficient details of methods and analysis provided to allow replication by others?

Yes

Reviewer Expertise:

Microbial infection and intestinal flora

I confirm that I have read this submission and believe that I have an appropriate level of expertise to confirm that it is of an acceptable scientific standard.

# Peer review: Reviewer response for version 1

**DOI:** 10.5256/f1000research.56059.r89070

**Published:** 2021-07-27

**Authors:** Kenichi Tamama

**Affiliations:** 1 Department of Pathology, University of Pittsburgh School of Medicine, Pittsburgh, PA, USA

The corresponding author has been very active in the research about auto-brewery syndrome (ABS) or gut fermentation syndrome. In this article, the authors tried to identify novel factors associated with ABS by conducting a survey-based case-control study for 46 ABS patients and their household members and comparing the survey results with a cohort of the American Gut Project participants (N=11,297).

 The article is well-written and the relevant articles are appropriately cited. Their data indicate the possible presence of allergic components in the ABS pathogenesis. This is a novel finding, but its significance is uncertain at the moment. This hypothesis should be further explored, as the authors state in the article.

Is the work clearly and accurately presented and does it cite the current literature?

Yes

If applicable, is the statistical analysis and its interpretation appropriate?

Yes

Are all the source data underlying the results available to ensure full reproducibility?

Yes

Is the study design appropriate and is the work technically sound?

Yes

Are the conclusions drawn adequately supported by the results?

Yes

Are sufficient details of methods and analysis provided to allow replication by others?

Yes

Reviewer Expertise:

Chemical pathology, clinical toxicology, laboratory medicine, stem cell biology

I confirm that I have read this submission and believe that I have an appropriate level of expertise to confirm that it is of an acceptable scientific standard.
